# Novel Thymoquinone Derivative TQFL28 Inhibits Triple-Negative Breast Cancer (TNBC) Invasiveness In Vitro and In Vivo

**DOI:** 10.3390/cimb47060412

**Published:** 2025-06-01

**Authors:** Jiayue He, Hui Zou, Chunli Wei, Jun Du, Ting Xiao, Ting Li, Ali El-Far, Jingliang Cheng, Junjiang Fu, Xiaoyan Liu

**Affiliations:** 1 Key Laboratory of Epigenetics and Oncology, The Research Center for Preclinical Medicine, Southwest Medical University, Luzhou 646000, China; hejieyue@swmu.edu.cn (J.H.); zouhui@hunnu.edu.cn (H.Z.); weichunli2015@swmu.edu.cn (C.W.); xiaoting@swmu.edu.cn (T.X.); 20220199120034@stu.swmu.edu.cn (T.L.); alih.elfar2024@swmu.edu.cn (A.E.-F.); jingliangc@swmu.edu.cn (J.C.); 2 Key Laboratory of Study and Discovery of Small Targeted Molecules of Hunan Province, School of Medicine, Hunan Normal University, Changsha 410013, China; 3 Department of Chemistry, Southwest Medical University, Luzhou 646000, China; dujun@swmu.edu.cn

**Keywords:** thymoquinone, derivative, TQFL28, anti-cancer, invasiveness, triple-negative breast cancer

## Abstract

Although thymoquinone (TQ) has been reported as an anti-tumor small molecule well investigated in numerous tumors. In this study, we designed and synthesized a novel TQ derivative, TQFL28, with a molecular formula of C_20_H_23_NO_2_. TQFL28 showed stronger cytotoxicity or anti-proliferative activities against triple-negative breast cancer (TNBC) cell lines (BT549, MDA-MB-231, or 4T1) than TQ but is lower in the normal mammary epithelial cells, MCF10A. TQFL28 exhibited lower IC_50_ values toward BT549 (38.78 ± 1.589) and MDA-MB-231 (39.63 ± 1.598) cells compared to TQ, indicating its efficacy for TNBC cytotoxicity. TQFL28 inhibited the growth, migration, and invasiveness of TNBC cells of 4T1 and BT549 in vitro and tumor progression and metastasis in a 4T1 allograft animal model in vivo. Moreover, TQFL28 presents lower toxicity than TQ in mice, showing a 7-day half-lethal dose (LD_50_) of 59.43 mg/kg (41.6–83.6, 95% confidence interval). Altogether, our study obtained. In addition, TQFL28 induced a significant reduction in tumor volumes in the mouse model in comparison to the vehicle group. TQFL28, a novel small molecule, has a superior inhibitory effect and lower toxicity on TNBC both in vitro and in vivo. Thus, TQFL28 might have potential as a therapeutic small molecule for breast cancer, especially in TNBC.

## 1. Introduction

Breast cancer (BC) incidence is highly increased worldwide, including triple-negative breast cancer (TNBC), which is an aggressive BC subtype with poor prognosis leading to deaths in women worldwide [1,2,3]. TNBC accounts for about 15–20% of all BC. BC chemotherapy includes three main strategies: neoadjuvant, adjuvant, and salvage treatments. In early-stage cases, it can lower the risk of recurrence by about 30% [4]. Neoadjuvant chemotherapy is especially effective as it shrinks tumors, enables breast-conserving surgeries, and allows previously inoperable tumors to become resectable while targeting micrometastatic lesions [5]. Another tool is immunotherapy because BC hinders activated T-cells from eliminating tumors due to inhibitory interactions like PD-1 and TIM-3, leading to T-cell exhaustion. This results in the limited effectiveness of immune checkpoint inhibitors as standalone therapies in advanced BC [6,7].

Moreover, malignant tumors, including TNBC, develop resistance to traditional chemotherapy. Among various cancer treatment strategies, targeted therapy using small molecules is so far the most effective for cancer treatment [8]. Bioactive components isolated from natural products or herbs have a long history of being used as drugs known for their anti-cancer, anti-diabetic, anti-inflammatory, antioxidant, anesthetic, and neuroprotective activities, among other activities [9]. Natural agent artemisinin (Qinghaosu) is considered an old gift in traditional Chinese medicine (TCM) [10]. However, TCM has not been fully recognized because of limited effects, unknown active compounds, poorly defined molecular mechanisms, or expensiveness. Therefore, finding natural drugs with high effectiveness and low toxicity is an urgent issue. Intriguingly, thymoquinone (TQ), a main active ingredient of *Nigella sativa* seeds, has been reported to have the potential to treat a variety of disorders, including BC [11,12]. TQ can inhibit cancer cell proliferation, migration, and invasiveness in various malignant tumors, including BC [13,14], prostate [15], bladder [16], lung [17], and colorectal [18] cancers. The cytotoxic effects were reported to be unleashed by TQ-bovine serum albumin nanoparticles on lung cancer cells [19]. Also, TQ-pectin beads have underscored the potential of an effective oral drug delivery system for targeted therapy in colorectal cancer [20]. The nanoparticles-TQ delivery could enhance targeted BC therapy [21,22]. Interestingly, Noor et al. [23] recently discovered a new therapeutic strategy to resistant BC by developing a nanoparticle TQ-PLGA-PF68, in which the incorporation of TQ into the PLGA-PEG and Pluronics F68 formulation preserved its bioactivities. Given its promising therapeutic properties, TQ has the potential to be a candidate for clinical treatments [24,25,26,27].

However, TQ has its limitations using the original formation. For example, the activation for TQ concentration (IC_50_) is reportedly higher than 165 µM in BC cells [28]. In addition, the LD_50_ in mice after intraperitoneal injection of TQ was determined to be 104.7 mg/kg, showing high toxicity in mice [29,30]. For this reason, we developed new TQ derivatives that could be new avenues for cancer treatment [30]. It is necessary to develop TQ derivatives with high efficacy, low toxicity, and more anti-tumor properties. In the current study, we aim to develop a novel TQ derivative, TQFL28, with a molecular formula of C_20_H_23_NO_2_. This derivative was successfully synthesized and showed antitumor activities against TNBC in vitro and in vivo models.

## 2. Materials and Methods

### 2.1. Ethical Approval

The animal study was carried out following the institutional animal care guidelines of the Southwest Medical University and approved by the university committee (approval code: 20160086, approval date: 9 March 2016; approval code: 20240715-038, approval date: 15 July 2024).

### 2.2. Reagents and Cell Culture

CCK8, fetal bovine serum (FBS), DMEM, and RPMI 1640 medium were obtained as previously reported [30]. MCF10A (normal mammary epithelial cell line) and triple-negative BC cell lines (4T1, BT549, and MDA-MB-231) were obtained from ATCC (American Type Culture Collection) as previously reported [30] and maintained in medium supplemented with 10% FBS at 37 °C with 5% CO_2_. The Annexin V/Propidium iodide (PI) staining kit was bought from BD Biosciences (San Jose, CA, USA). The BALB/c mice were purchased from Tengxin Biotechnology Co., Ltd. (Beijing, China).

### 2.3. Chemical Synthesis

Chemical synthesis is described as previously reported [30]. TQ was purchased from Sigma-Aldrich (St. Louis, MO, USA). Sodium azide (NaN_3_) was purchased from Xiya Reagent Co. (Linyi, China). The 4-isopropylbenzaldehyde was purchased from the Sigma Co., Ltd. (Shanghai, China). Hydrochloric acid (HCl, analytical grade) was purchased from the Zhuzhou Quartz Glass Co., Ltd. (Zhuzhou, China), and anhydrous ethyl alcohol (EtOH) was obtained from the Sinopharm Group Chemical Reagent Co., Ltd. (Shanghai, China).

The experiments for ^1^H and ^13^C NMR were performed by a Bruker AV-400 MHz spectrometer (Bruker, Karlsruhe, Germany) using tetramethylsilane as an internal standard. High-resolution mass spectrometry (HR-MS) was performed using electrospray ionization (ESI) and the quadrupole tandem time-of-flight (QTOF) mass analyzer (X500R, AB Sciex; Framingham, MA, USA).

The obtained TQFL28 is a yellow solid with a yield of 253 mg (81.9%) and was subjected to ^1^H and ^13^C NMR identification. Results for ^1^H NMR (400 MHz, DMSO-*d*_6_) *δ* 9.18 (s, 1H), 8.07 (d, *J* = 8.1 Hz, 2H), 7.42 (d, *J* = 8.1 Hz, 2H), 6.75 (s, 1H), 3.29–3.19 (m, 1H), 2.99–2.89 (m, 1H), 2.34 (s, 3H), 1.33 (d, *J* = 7.0 Hz, 6H), 1.21 (d, *J* = 7.0 Hz, 6H) (Figure S1A). Results for ^13^C NMR (101 MHz, DMSO-*d*_6_) *δ* 161.7, 152.2, 152.1, 141.8, 141.6, 128.1, 127.1, 127.1, 124.7, 111.6, 110.4, 38.9, 33.5, 28.9, 23.5, 22.5 (Figure S1B). High-resolution mass spectrum (HRMS) (*m*/*z*, ESI) calcd for C_20_H_24_NO_2_ [M+H]^+^: 310.1802; found 310.1809 (Figure S2).

### 2.4. Cell Counting Kit-8 (CCK8) Assays

Cells were seeded in a 96-well plate with a concentration of 3000–5000 cells per well and treated with various concentrations of TQ or TQFL28 for 24 h and 48 h treatments, respectively. After the treatments, 10 µL of CCK8 reagents were supplemented and incubated at 37 °C for 2 h. Then, the absorbance at 450 nm was monitored by the microplate reader [31]. IC_50_ values were calculated using GraphPad Prism 9 software (San Diego, CA, USA) to draw the dose–effect curves. All experiments were repeated three times.

### 2.5. Assays for Cell Growth, Migration, and Invasion

A real-time cell analyzer (RTCA) was used for cell growth, migration, and invasion analyses. The 16-well E-plates were applied to cell growth with 100 µL of cells (1 × 10^4^ cells/mL) in each well. CMI plates for RTCA with an electronically integrated Boyden Chamber were applied to analyze cell migration and invasion index, and the lower chambers were filled with chemotaxis inducer (10% serum-supplemented media), and the upper chambers contained additional cell suspensions (1 × 10^4^ cells/mL). For the cell invasion assay, Matrigel in 1 × PBS was added to the CMI plates. After 8 h of cell growth, 5 or 10 µM of TQFL28 or vehicle (DMSO) was added. The cell growth, migration, and invasion were monitored by an RTCA instrument (xCELLigence RTCA DP; Roche, Mannheim, Germany) [32]. All experiments were repeated three times.

### 2.6. Apoptosis and Cell Cycle Assays

Assays for apoptosis and cell cycle were conducted as previously described [31], using BT549, 4T1, or MDA-MB-231 cells, which were treated with TQFL28 at concentrations of 0, 2.5, or 5 μM [30,31]. For details on cell cycle detection, cells were collected, resuspended in 500 µL pre-cooled 70% ethanol, and fixed overnight at 4 °C. The cells were centrifuged at 500× *g* for 5 min, and the supernatants were discarded. The cell pellets were washed once with 1 × PBS, 500 μL of PI/RNase Staining Buffer (BD, cat #: 550825, Franklin Lakes, NJ, USA) staining solution, incubated for 30 min in the dark, and used by flow cytometry (BD FACSVerse™ Cell Analyzer, Franklin Lakes, NJ, USA) to detect cell cycle distribution (G0/G1 phase, S phase, and G_2_/M phase). The Annexin V-FITC/PI double staining method was used for apoptosis detection: First, cells were resuspended, and the cell concentration was adjusted to 1 × 10^6^ per mL; then, 100 μL of cell suspension was taken, and 5 μL of Annexin V-FITC (BD, cat #: 556547, Franklin Lakes, NJ, USA) and 5 μL of PI were sequentially added, gently mixed, and incubated in the dark at room temperature for 15–20 min. After incubation, the same flow cytometry was used to detect and analyze cell apoptosis. All experiments were repeated three times.

### 2.7. Mouse Allograft Assays

To set up the BC allograft model, a 4T1 cell line was injected into the mammary fat pads of female BALB/c mice (*n* = 18), and the size of the tumors was monitored every 5 days [32]. Four days after 4T1 cell injections, the mice were randomly allocated into three groups, six in each group, and supplemented with 0, 3, and 7.5 mg/kg of TQFL28, respectively. The tumor sizes were continuously measured following Equation (1), where V is the tumor size, L is the tumor length, and W is the tumor width.

(1) $$V=\frac{L\times W^{2}}{2}$$

At the end of the 30-day injections (27-day TQFL28 treatment), the mice were sacrificed, the tumor tissues were dissected, and the tumor weights were calculated. To evaluate the TQFL28 and TQ effects on tumor cell migration and invasion, the mice’s lungs were dissected at the end of the treatment, and the tumor colonies were counted. In addition, the tumor lengths in percentages to control were measured using the Image J analysis software (Image J v1.46r, National Institute of Health, Bethesda, MD, USA).

### 2.8. Histopathological Assessment

Tissues for tumors or the lungs were fixed in 4% paraformaldehyde for 24 h, embedded in paraffin, and sliced to 5 µm thickness. After dewaxing and hydrating in a xylene and alcohol series, the slides were stained with H&E (hematoxylin and eosin) and then dipped into the other alcohol and xylene series to dehydrate. A coverslip with one or two drops of neutral gum was used to cut off the air and cover the slides, and photos were taken [32].

### 2.9. Mouse Toxicity Assays

The BALB/c mice, around 8 weeks of age, were selected, half male and half female. Eight mice were in each experimental group and each control group. Intraperitoneal injections of 0, 25, 50, 80, and 120 mg/kg of TQFL28 or TQ were used for each group. The general health and death of mice were observed and recorded at 2, 6, 9, and 24 h on the first day of TQFL28 or TQ administration. Treated mice were observed once every 24 h, and the data were recorded. Using the Bliss method, the collected data were calculated by the IBM SPSS Statistics for Windows, version 20 (IBMCorp., Armonk, NY, USA) to determine the half-lethal dose (LD_50_) of TQFL28 and TQ. The LD_50_ was measured following Equation (2), where Xm is the logarithm of the maximum dose, p is the animal mortality rate, Σp is the sum of mortality rates across all groups, and I is the logarithm of the ratio between two adjacent doses (higher dose as the numerator).

(2) LD50=log−1[Xm−I(Σp−0.5)]

### 2.10. Statistical Analysis

The statistical differences were conducted by one-way ANOVA using the GraphPad Prism 6, which was described previously [30]. *p* value < 0.05 was considered significantly different. * *p* < 0.05 and ** *p* < 0.01 are indicated as differences with *p* values.

## 3. Results

### 3.1. Synthesis of a New TQ Derivative TQFL28

**Figure 1:**
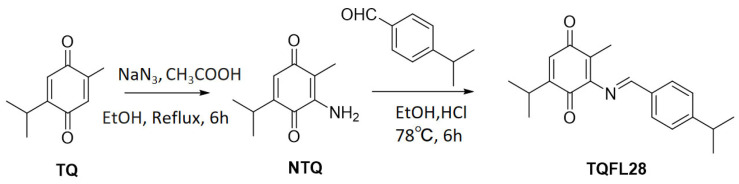
The synthetic route and structure for TQFL28. NTQ is 3-amino-5-isopropyl-2-methylcyclohexa-2,5-diene-1,4-dione [30]. TQFL28 is (E)-5-isopropyl-3-((4-isopropylbenzylidene)amino)-2-methylcyclohexa-2,5-diene-1,4-dione. The molecular formula for TQFL28 is C_20_H_23_NO_2_. We developed the TQFL28 small molecule by adding a side chain at C-6 of TQ, since synthesized derivatives showed that the compounds with unsaturated side chains confer higher activities than those with equally long saturated chains. NaN_3_ is sodium azide.

It was reported that compounds’ derivatives with unsaturated side chains conferred higher activities than those with equally long saturated chains [33]. With TQ as the starting material, we synthesized a new TQ derivative, TQFL28 (molecular formula: C_20_H_23_NO_2_), by adding a side chain at the number of the carbon where the side chain was added. Two steps were used to achieve it. In the first step, the intermediate NTQ (3-amino-5-isopropyl-2-methylcyclohexa-2,5-diene-1,4-dione) was synthesized, referred to in our previously published paper [30]. Then, a mixture of NTQ (0.179 g, 1 mM) and 4-isopropylbenzaldehyde (0.148 g, 1 mM) in EtOH (20 mL) with HCl (0.5 mL) was heated and stirred at 78 °C for 6 h. Then, the resulting mixture was filtered to obtain the filtrate. The filtrate was concentrated under reduced pressure conditions and ran on a silica gel column using a mixture of petroleum ether and ethyl acetate in a 30:1 ratio as an eluent to collect the purified TQFL28 product: (E)-5-isopropyl-3-((4-isopropylbenzylidene)amino)-2-methylcyclohexa-2,5-diene-1,4-dione (Figure 1).

### 3.2. TQFL28 Shows Higher Cytotoxic Sensitivity than TQ in Breast Cancer Cells

**Figure 2:**
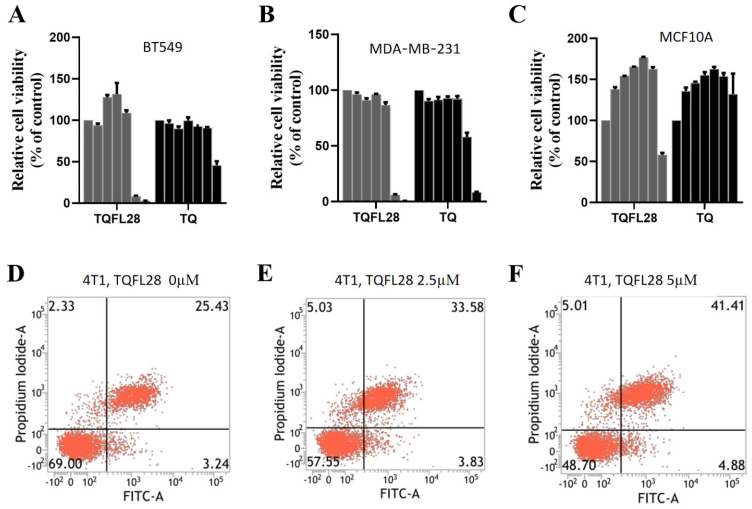
Effects of TQFL28 and TQ on the viability of different breast cancer cells and normal breast epithelial cells. (**A**,**B**) CCK8 assays show the effect of TQFL28 and TQ at 24 h in BT549 (**A**) and MDA-MB-231 (**B**), respectively. (**C**) The effect of TQFL28 and TQ on 24 h in MCF10A (normal breast epithelial cell line). (**D**–**F**) The apoptotic effect of TQFL28 in 4T1 at the indicated TQFL28 concentrations. A total of 0 µM indicates no TQFL28 treatments. The results are presented as the mean ± SD (*n* = 3).

To know whether TQFL28 has cytotoxic effects higher than TQ, we analyzed their cytotoxic effects on TNBC cell lines. We treated two TNBC cells, BT549 and MDA-MB-231, with TQFL28 and TQ at different concentrations at 0, 2.5, 5, 10, 20, 40, and 80 μM and performed CCK8 assays for cell viability. The results are shown in both Figure 2A,B and Table 1; we found that TQFL28’s cytotoxic effects were time-dependent on these two cell lines. Moreover, we found that the cytotoxic sensitivity of TQFL28 is higher than TQ on these cells. The IC_50_ values of TQFL28 were 38.78 for BT549 and 39.63 for MDA-MB-231 at 24 h, while they were 27.32 for BT549 and 27.53 for MDA-MB-231 at 48 h, respectively. However, the IC_50_ values of TQ were 63.67 for BT549 and 57.17 for MDA-MB-231 at 24 h and 64.15 for BT549 and 40.85 for MDA-MB-231 at 48 h, respectively. To further elucidate whether the toxic effect of TQFL28 is cancer-specific, we compared the IC_50_ of TQFL28 on the MCF10A cell line with TNBC cell lines. The results for MCF10A viability are shown in Table 1 and Figure 2C, and the IC_50_ for MCF10A is higher than that of all tested TNBC cell lines, respectively. These results revealed that the cytotoxic effects of TQFL28 are more sensitive than TQ, which are specific to BC cell lines.

### 3.3. Effect on Cell Apoptosis by TQFL28

Given that TQFL28 treatment affected cytotoxicity in TNBC cells, we examined its pro-apoptotic roles by flow cytometry in 4T1 and BT549. The results showed that TQFL28 had almost no effect on the cell cycle in both cells but significantly affected apoptosis in 4T1 cells with 2.5 µM of TQFL28 treatment; more effects appeared when treated using 5 µM of TQFL28 (Figure 2E,F, *p* < 0.05). In addition, no effect was found on apoptosis in BT549 cells. Further investigation could be conducted in the future.

### 3.4. TQFL28 Suppresses the Growth, Migration, and Invasiveness of Breast Cancer Cells

**Figure 3:**
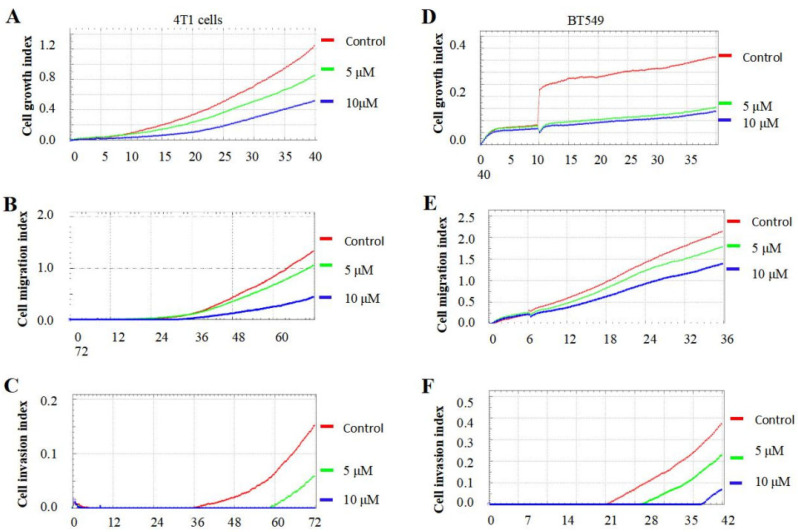
TQFL28 suppresses breast cancer cell growth, migration, and invasion. (**A**–**C**) TQFL28 suppresses breast cancer cell growth (**A**), migration (**B**), and invasion (**C**) in the indicated concentrations of 4T1 cells. (**D**–**F**) TQFL28 suppresses breast cancer cell growth (**D**), migration (**E**), and invasion (**F**) in the indicated concentrations of BT549 cells. The Y-axis indicates the cell index (cell numbers), while the X-axis indicates the treated time (h).

TQ was reported to inhibit BC cell growth, migration, and invasiveness [13]. To know the specific effects of TQFL28, we performed cancer cell growth, migration, and invasion in 4T1 and BT549 cells using a real-time cell analyzer. We checked the TQFL28 effect for different concentrations (0, 5, and 10 μM). We also used a concentration of 10 μM for TQFL28, which we used previously for TQ [13]. The growth, migration, and invasion cell indices showed that TQFL28 remarkably suppresses cancer cell growth (Figure 3A,D), migration (Figure 3B,E), and invasion (Figure 3C,F) at 5 and 10 μΜ for 4T1 and BT549 cell lines, respectively. Moreover, these specific inhibitions are presented in a dose-dependent manner. Thus, this study concludes that TQFL28 inhibits growth, migration, and invasion in TNBC cells.

### 3.5. TQFL28 Inhibits Tumor Growth in Mice

**Figure 4:**
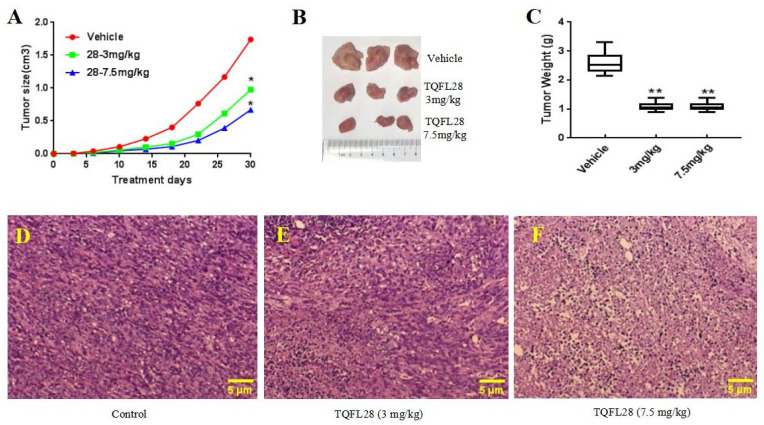
Effect of TQFL28 on breast cancer tumor growth in mice’s allograft. (**A**) TQFL28 suppresses tumor growth with the tumor size in a dose-dependent manner. (**B**) TQFL28 suppresses tumor growth measured with the tumor weights in a dose-dependent manner. (**C**) The quantitative data for (**B**). Representative images for in situ tumors without (**D**) or with (**E**,**F**) TQFL28 treatments (0, 3, or 7.5 mg/kg, respectively). “*” indicates *p* < 0.05 and “**” indicates *p* < 0.01. Vehicle: the vehicle-treated group. Scale bar = 5 μM.

4T1 is a TNBC cell line from the mouse. To know whether TQFL28 can inhibit BC cell growth, migration, and invasion in mice, we generated mouse tumor allograft models using the 4T1 TNBC cell line. Data are in Figure 4A. We revealed that compared to the control group, the tumor sizes in situ were remarkably decreased by TQFL28 treatment in a dose-dependent fashion. Moreover, after sacrificing mice on the 30th day, we found that the tumor weights were also significantly reduced by TQFL28 treatment in a dose-dependent fashion compared to the vehicle-treated group (Figure 4B,C). As shown in our previous study, high-concentration TQ was very toxic. Then, we checked the morphology changes of in situ tumors by H&E staining. Results are shown in Figure 4D–F. We revealed that the tissue morphologies of the tumors were remarkably changed by TQFL28 treatment in a dose-dependent manner compared to the vehicle-treated group, including cell shrinking and nuclear condensing (karyopyknosis), as a pro-apoptosis status, particularly at high dosage (7.5 mg/kg) of TQFL28 treatment (Figure 4F).

### 3.6. TQFL28 Inhibits Migration to the Lungs of Breast Cancer Cells In Vivo

**Figure 5:**
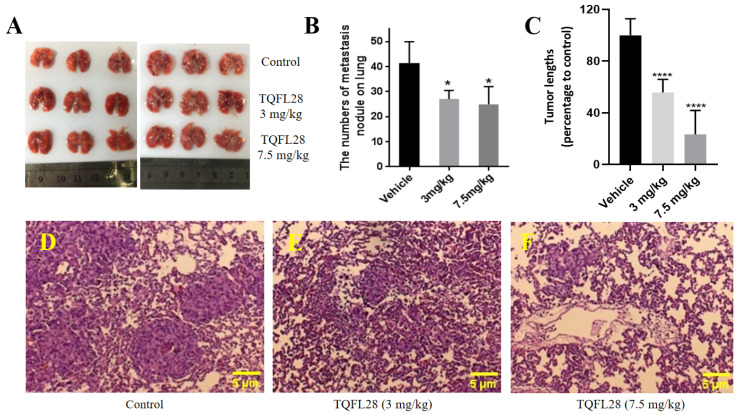
TQFL28 treatment suppresses breast cancer metastasis to the lungs. (**A**) The size of metastasis colonies on the lungs was measured in TQFL28 treatment with 0 mg/kg, 3 mg/kg, or 7.5 mg/kg, respectively. (**B**) The number of metastasis nodules on the lungs after TQFL28 treatment. (**C**) The tumor length on the lungs after TQFL28 treatment (percentages to control). (**D**) Representative images for metastatic tumors on the lungs without TQFL28 treatment. (**E**) Representative images for lung metastatic tumors by 3 mg/kg TQFL28 treatments. (**F**) Representative images for lung metastatic tumors by 7.5 mg/kg TQFL28 treatments. “*” indicates *p* < 0.05 and “****” indicates *p* < 0.0001. Scale bar = 5 μM.

Using the 4T1 cell-derived allograft model, we then examined the effects of TQFL28 treatment on tumor cell migration and invasion as evaluated by the number of colonies in the lungs. Figure 5A showed numerous colonies formed in the lungs of the control group (vehicle-treated, TQFL28 0 mg/kg), whereas only a few colonies were found in the TQFL28-treated groups. Furthermore, the overall sizes of the colonies in the vehicle-treated group are remarkably bigger than those in the TQFL28-treated groups. The quantitative data of the average number of colonies per group are presented in Figure 5B. The tumor length on the lungs after TQFL28 treatment (percentages to control) is in Figure 5C. It revealed a significant reduction in lung metastatic nodules in a dose-dependent fashion. Interestingly, images of tumors in the lungs treated with TQFL28 showed smaller tumor sizes compared to those of the vehicle-treated group (Figure 5D–F). Thus, our in vivo data unambiguously supported that TQFL28 significantly inhibited BC cell migration and invasion.

### 3.7. TQFL28 Shows Less Toxicity in Mice than TQ

We next sought to know the toxicity in mice for TQFL28 and TQ. The data are shown in Table 2. The high-dose group of the TQFL28 group completely died in one week, and the rest of the groups showed concentration-dependent mortality, with mortality of 0, 12.5, 25, and 62.5%, respectively, within one week. However, all the concentration gradient mice in the TQ group died within 48 h, with different degrees of reduced activity, including hunchback, walking instability, dyspnea, and other manifestations, and the mortality rate within 24 h in each dose group was 0%, 25%, 87.5%, 87.5%, and 100%, respectively, in a concentration-dependent increase. After calculation, the 24 h half-lethal dose (LD_50_) of TQ was 33.75 mg/kg with a 17.5–46.5 95% confidence interval (CI), while no deaths were recognized in TQFL28. In addition, at 7 days the half-lethal dose (LD_50_) of TQFL28 was 59.43 mg/kg with 41.6–83.6, 95% CI. Thus, TQFL28 showed significantly lower toxicity in mice than TQ. Of course, there was no long-term toxicity assessment; future studies should be conducted on that.

## 4. Discussion

TQ is the main active ingredient derived from the seeds of *Nigella sativa*. TQ has been reported to have the potential for treating a variety of disorders, including BC [11,12,34,35,36,37] and other cancers [38]. However, TQ has limitations, such as toxicity in mice at high concentrations. It was reported that compounds’ derivatives with unsaturated side chains conferred higher activities than those with equally long saturated chains [33]. In our efforts to improve the anticancer properties of TQ, we have successfully developed the TQFL28 by adding the side chain at C-6 of TQ. This innovative derivative demonstrates a significantly enhanced anticancer effect compared to TQ with more safety. Firstly, TQFL28 showed more potent cytotoxicity or anti-proliferative effects against TNBC cells than TQ but was low in the normal mammary epithelial cell line, MCF10A. The IC_50_ values of TQFL28 were 38.78 for BT549 and 39.63 for MDA-MB-231 at 24 h and 27.32 for BT549 and 27.53 for MDA-MB-231 at 48 h, showing around 1-fold lower cytotoxicity than TQ. Secondly, TQFL28 inhibited growth, migration, and invasion in TNBC cells and reduced the tumor growth and metastasis in 4T1 cancer cell-derived allograft animal models. Of course, future studies using more BC cell lines, even other types of cell lines, should be conducted in the future. Unfortunately, it is still missing control groups, specifically the unmodified TQ in the in vivo experiments; we cannot definitively conclude whether the observed enhancement in tumor inhibition is attributable to the structural modifications made.

TQ has demonstrated a wide array of biological activities, which have led researchers to conduct several studies to determine its LD_50_ for in vivo applications. This critical evaluation helps to understand the safe dosage levels of TQ when applied in living organisms, ensuring that its therapeutic potential can be harnessed effectively while minimizing risks. In the current study, TQ has an LD_50_ of 33.75 mg/kg at 24 h of intraperitoneal injection, while TQFL28 exhibited an LD_50_ of 59.43 mg/kg at 168 h. Al-Ali et al. [29] reported that the LD_50_ of TQ in mice was 104.7 mg/kg with 89.7–119.7, 95% CI for intraperitoneal injection. Mansour et al. [39] reported that the LD_50_ of TQ in mice was 90.3 mg/kg with 77.9–104.7, 95% CI. Our results showed the LD_50_ of TQ was 33.75 mg/kg with 17.5–46.5, 95% CI, with more toxicity than the above-mentioned reports; this difference might be due to different vehicles or methods used to estimate the LD_50_. We should point out that we did subacute toxicity experiments with 7 days for LD_50_ of 59.43 mg/kg, which can’t compare with TQ with 24 h for LD_50_ of 33.75 mg/kg. Nevertheless, our TQFL28 exhibited lower toxicity than TQ in vivo. Taken together, TQFL28 could possess better inhibitory effects on TNBC both in vitro and in vivo but lower toxicity on normal cells/animals.

As an aggressive subtype with a poor prognosis in BC, TNBC continues to be a leading cause of cancer-related death in females worldwide. Since TQ processes potentials on human health and patients with BC, we conclude that TQFL28 would also have inhibitory effects for TNBC. Further studies should be conducted on the mechanisms of TQFL28 in inhibiting the growth, migration, and invasion of BC cells.

## 5. Conclusions

In conclusion, our research has unveiled an innovative small molecule, TQFL28, which shows a notably greater inhibitory effect on TNBC while presenting reduced toxicity levels in both in vitro and in vivo studies. These encouraging results indicate that TQFL28 possesses significant potential as a therapeutic agent for BC, especially in tackling the challenges associated with TNBC. Moreover, additional investigations are required to understand the mechanistic anticancer function of TQFL28 against TNBC thoroughly.

## 6. Patents

The authors have stated that no conflicts of interest exist, except for a patent (cat #: ZL 2021 1 0159546.1) granted.

## Figures and Tables

**Table 1:** The IC_50_ values of TQFL28 and TQ against breast cancer cells.

	IC_50_ Values at 24 h (μM)		IC_50_ Values at 48 h (μM)	
Cell lines	TQFL28	TQ	TQFL28	TQ
BT549	38.78 ± 1.589	63.67 ± 1.804	27.32 ± 1.436	64.15 ± 1.807
MDA-MB-231	39.63 ± 1.598	57.17 ± 1.757	27.53 ± 1.440	40.85 ± 1.611
MCF-10A	74.20 ± 1.870	>100	43.30 ± 1.636	>100

**Table 2:** Half-lethal doses (LD_50_) of TQ and TQFL28 treatments in mice.

	D\H	24	48	72	96	120	144	168
TQ	120	8	8	/	/	/	/	/
(mg/kg)	80	7	8	/	/	/	/	/
	50	7	8	/	/	/	/	/
	25	2	8	/	/	/	/	/
	LD_50_	33.75						
	95% IC	17.5–46.5						
TQFL28	120	0	4	8	8	8	8	8
(mg/kg)	80	0	3	3	5	5	5	5
	50	0	1	1	1	1	2	2
	25	0	1	1	1	1	1	1
	LD_50_							59.43
	95% IC							41.6–83.6

Note: Each group was eight mice. IC, interval confidence; D, dosage; H, hours.

## Data Availability

The original contributions presented in this study are included in the article. Further inquiries can be directed to the corresponding authors.

## Supplementary Materials

The following supporting information can be downloaded at: https://www.mdpi.com/article/10.3390/cimb47060412/s1.

## Abbreviations

TQ	Thymoquinone
TQFL28	(E)-5-isopropyl-3-((4-isopropylbenzylidene)amino)-2-methylcyclo hexa-2,5-diene-1,4-dione
TNBC	Triple-negative breast cancer
TCM	Traditional Chinese medicine
PI	Propidium iodide
CCK8	Cell Counting Kit-8
NMR	Nuclear magnetic resonance
HRMS	High-resolution mass spectrum
LD_50_	Half-lethal dose
IC_50_	Half maximal inhibitory concentration
RTCA	Real-time cell analyzer

## References

[1] DeSantis C.E., Ma J., Gaudet M.M., Newman L.A., Miller K.D., Goding Sauer A., Jemal A., Siegel R.L. (2019). Breast cancer statistics, 2019. CA Cancer J. Clin..

[2] Siegel R.L., Miller K.D., Jemal A. (2020). Cancer statistics, 2020. CA Cancer J. Clin..

[3] Cheng J., Song B., Wei C., Zhang L., Liu X., Yang L., Tima S., Chiampanichayakul S., Xiao X., Anuchapreeda S. (2025). Exploring breast cancer associated-gene panel for next-generation sequencing and identifying new, pathogenic variants in breast cancer from western China. J. Cancer.

[4] Wang J., Wu S.G. (2023). Breast Cancer: An Overview of Current Therapeutic Strategies, Challenge, and Perspectives. Breast Cancer.

[5] Mano M.S., Awada A. (2004). Primary chemotherapy for breast cancer: The evidence and the future. Ann. Oncol..

[6] Warwas K.M., Meyer M., Goncalves M., Moldenhauer G., Bulbuc N., Knabe S., Luckner-Minden C., Ziegelmeier C., Heussel C.P., Zornig I. (2021). Co-Stimulatory Bispecific Antibodies Induce Enhanced T Cell Activation and Tumor Cell Killing in Breast Cancer Models. Front. Immunol..

[7] Joyce J.A., Fearon D.T. (2015). T cell exclusion, immune privilege, and the tumor microenvironment. Science.

[8] Jazieh K., Bell R., Agarwal N., Abraham J. (2020). Novel targeted therapies for metastatic breast cancer. Ann. Transl. Med..

[9] Zhao Z., Jia Q., Wu M.S., Xie X., Wang Y., Song G., Zou C.Y., Tang Q., Lu J., Huang G. (2018). Degalactotigonin, a Natural Compound from Solanum nigrum L., Inhibits Growth and Metastasis of Osteosarcoma through GSK3beta Inactivation-Mediated Repression of the Hedgehog/Gli1 Pathway. Clin. Cancer Res. An. Off. J. Am. Assoc. Cancer Res..

[10] Tu Y. (2011). The discovery of artemisinin (qinghaosu) and gifts from Chinese medicine. Nat. Med..

[11] Ahmad A., Mishra R.K., Vyawahare A., Kumar A., Rehman M.U., Qamar W., Khan A.Q., Khan R. (2019). Thymoquinone (2-Isoprpyl-5-methyl-1, 4-benzoquinone) as a chemopreventive/anticancer agent: Chemistry and biological effects. Saudi Pharm. J. SPJ Off. Publ. Saudi Pharm. Soc..

[12] Noor N.S., Kaus N.H.M., Szewczuk M.R., Hamid S.B.S. (2021). Formulation, Characterization and Cytotoxicity Effects of Novel Thymoquinone-PLGA-PF68 Nanoparticles. Int. J. Mol. Sci..

[13] Khan M.A., Tania M., Wei C., Mei Z., Fu S., Cheng J., Xu J., Fu J. (2015). Thymoquinone inhibits cancer metastasis by downregulating TWIST1 expression to reduce epithelial to mesenchymal transition. Oncotarget.

[14] Unal T.D., Hamurcu Z., Delibasi N., Cinar V., Guler A., Gokce S., Nurdinov N., Ozpolat B. (2021). Thymoquinone Inhibits Proliferation and Migration of MDA-MB-231 Triple Negative Breast Cancer Cells by Suppressing Autophagy, Beclin-1 and LC3. Anti-Cancer Agents Med. Chem..

[15] Kaseb A.O., Chinnakannu K., Chen D., Sivanandam A., Tejwani S., Menon M., Dou Q.P., Reddy G.P. (2007). Androgen receptor and E2F-1 targeted thymoquinone therapy for hormone-refractory prostate cancer. Cancer Res..

[16] Zhang M., Du H., Huang Z., Zhang P., Yue Y., Wang W., Liu W., Zeng J., Ma J., Chen G. (2018). Thymoquinone induces apoptosis in bladder cancer cell via endoplasmic reticulum stress-dependent mitochondrial pathway. Chem.-Biol. Interact..

[17] Samarghandian S., Azimi-Nezhad M., Farkhondeh T. (2019). Thymoquinone-induced antitumor and apoptosis in human lung adenocarcinoma cells. J. Cell. Physiol..

[18] Gali-Muhtasib H., Diab-Assaf M., Boltze C., Al-Hmaira J., Hartig R., Roessner A., Schneider-Stock R. (2004). Thymoquinone extracted from black seed triggers apoptotic cell death in human colorectal cancer cells via a p53-dependent mechanism. Int. J. Oncol..

[19] Durga B.B., Ramachandran V., Senthil B., Soloman V.G., Elshikh M.S., Almutairi S.M., Wen Z.H., Lo Y.H. (2024). Unleashing of cytotoxic effects of thymoquinone-bovine serum albumin nanoparticles on A549 lung cancer cells. Open Life Sci..

[20] Alfatama M., Choukaife H., Al Rahal O., Zin N.Z.M. (2024). Thymoquinone Pectin Beads Produced via Electrospray: Enhancing Oral Targeted Delivery for Colorectal Cancer Therapy. Pharmaceutics.

[21] Arvejeh P.M., Chermahini F.A., Soltani A., Lorigooini Z., Rafieian-Kopaei M., Mobini G.R., Khosravian P. (2024). Improved Therapeutic Efficacy: Liposome-Coated Mesoporous Silica Nanoparticles Delivering Thymoquinone to MCF-7 Cells. Curr. Drug Deliv..

[22] Mehanna M.M., Sarieddine R., Alwattar J.K., Chouaib R., Gali-Muhtasib H. (2020). Anticancer Activity of Thymoquinone Cubic Phase Nanoparticles Against Human Breast Cancer: Formulation, Cytotoxicity and Subcellular Localization. Int. J. Nanomed..

[23] Noor N.S., Hamid S.B.S. (2025). Thymoquinone-PLGA-PF68 Nanoparticles Induce S Phase Cell Cycle Arrest and Apoptosis, Leading to the Inhibition of Migration and Colony Formation in Tamoxifen-Resistant Breast Cancer Cells. Curr. Mol. Med..

[24] Abukhader M.M. (2013). Thymoquinone in the clinical treatment of cancer: Fact or fiction?. Pharmacogn. Rev..

[25] Barkat M.A., Harshita, Ahmad J., Khan M.A., Beg S., Ahmad F.J. (2018). Insights into the Targeting Potential of Thymoquinone for Therapeutic Intervention Against Triple-negative Breast Cancer. Curr. Drug Targets.

[26] Khan M.A., Tania M., Fu S., Fu J. (2017). Thymoquinone, as an anticancer molecule: From basic research to clinical investigation. Oncotarget.

[27] Tiwari G., Gupta M., Devhare L.D., Tiwari R. (2024). Therapeutic and Phytochemical Properties of Thymoquinone Derived from Nigella sativa. Curr. Drug Res. Rev..

[28] Bashmail H.A., Alamoudi A.A., Noorwali A., Hegazy G.A., Ajabnoor G.M., Al-Abd A.M. (2020). Thymoquinone Enhances Paclitaxel Anti-Breast Cancer Activity via Inhibiting Tumor-Associated Stem Cells Despite Apparent Mathematical Antagonism. Molecules.

[29] Al-Ali A., Alkhawajah A.A., Randhawa M.A., Shaikh N.A. (2008). Oral and intraperitoneal LD_50_ of thymoquinone, an active principle of Nigella sativa, in mice and rats. J. Ayub Med. Coll. Abbottabad.

[30] Wei C., Zou H., Xiao T., Liu X., Wang Q., Cheng J., Fu S., Peng J., Xie X., Fu J. (2021). TQFL12, a novel synthetic derivative of TQ, inhibits triple-negative breast cancer metastasis and invasion through activating AMPK/ACC pathway. J. Cell. Mol. Med..

[31] Wei C., Yao X., Jiang Z., Wang Y., Zhang D., Chen X., Fan X., Xie C., Cheng J., Fu J. (2019). Cordycepin Inhibits Drug-resistance Non-small Cell Lung Cancer Progression by Activating AMPK Signaling Pathway. Pharmacol. Res..

[32] Fu J., Qin L., He T., Qin J., Hong J., Wong J., Liao L., Xu J. (2011). The TWIST/Mi2/NuRD protein complex and its essential role in cancer metastasis. Cell Res..

[33] Breyer S., Effenberger K., Schobert R. (2009). Effects of thymoquinone-fatty acid conjugates on cancer cells. ChemMedChem.

[34] Sadeghi E., Imenshahidi M., Hosseinzadeh H. (2023). Molecular mechanisms and signaling pathways of black cumin (*Nigella sativa*) and its active constituent, thymoquinone: A review. Mol. Biol. Rep..

[35] Pandey R., Natarajan P., Reddy U.K., Du W., Sirbu C., Sissoko M., Hankins G.R. (2025). Deciphering the dose-dependent effects of thymoquinone on cellular proliferation and transcriptomic changes in A172 glioblastoma cells. PLoS ONE.

[36] Qiu R.B., Zhao S.T., Xu Z.Q., Hu L.J., Zeng R.Y., Qiu Z.C., Peng H.Z., Zhou L.F., Cao Y.P., Wan L. (2025). Thymoquinone mitigates cardiac hypertrophy by activating adaptive autophagy via the PPAR-gamma/14-3-3gamma pathway. Int. J. Mol. Med..

[37] Shrief A.I., Elshenawy D.S., Elsukary A.E., Elekhtiar S.A., Yahia O.A. (2025). Behavioral and histological study on the neuroprotective effect of thymoquinone on the cerebellum in AlCl3-induced neurotoxicity in rats through modulation of oxidative stress, apoptosis, and autophagy. J. Mol. Histol..

[38] Gurbilek M., Deniz C.D., Eroglu Gunes C., Kurar E., Reisli I., Kursunel M.A., Topcu C., Koc M. (2025). Anticancer activity of thymoquinone in non-small cell lung cancer and possible involvement of PPAR-gamma pathway. Int. J. Radiat. Biol..

[39] Mansour M.A., Ginawi O.T., El-Hadiyah T., El-Khatib A.S., Al-Shabanah O.A., Al-Sawaf H.A. (2001). Effects of volatile oil constituents of Nigella sativa on carbon tetrachloride-induced hepatotoxicity in mice: Evidence for antioxidant effects of thymoquinone. Res. Commun. Mol. Pathol. Pharmacol..

