# Supplementary material for: Novel Thymoquinone Derivative TQFL28 Inhibits Triple-Negative Breast Cancer (TNBC) Invasiveness In Vitro and In Vivo
Source: Curr Issues Mol Biol. 2025 Jun 1;47(6):412. doi: 10.3390/cimb47060412 (PMC12191647; doi:10.3390/cimb47060412)
Supplement: Supplementary file 1 [file cimb-47-00412-s001.zip › cimb-3629503-supplementary.pdf]

Supplementary Materials

Supplemmentary Figure S1

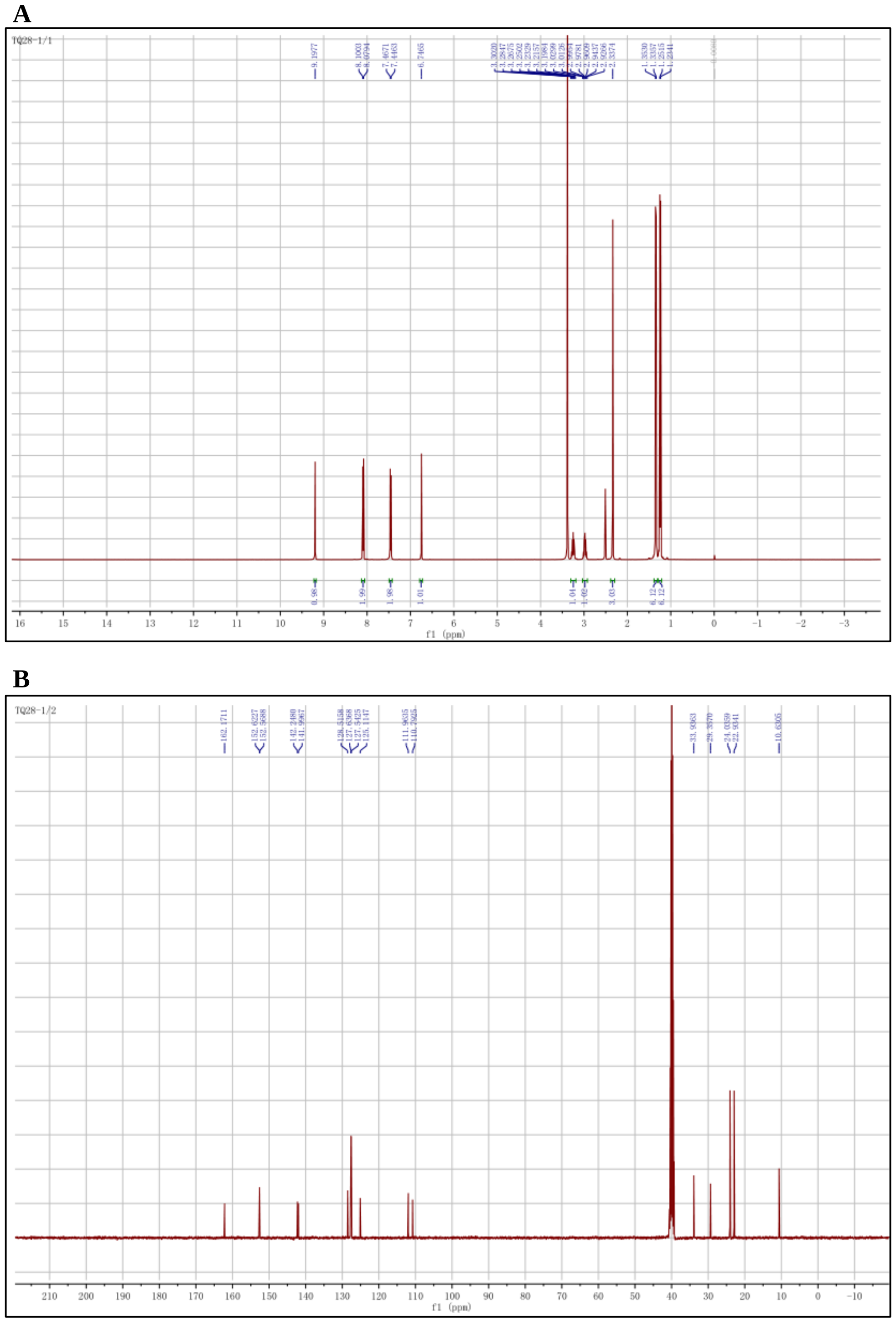

Figure S1. The nuclear magnetic resonance (NMR) for TQFL28. A. ^{1}H NMR spectrum of TQFL28. B. ^{13}C NMR spectrum of TQFL28.

Supplemmentary Figure S2

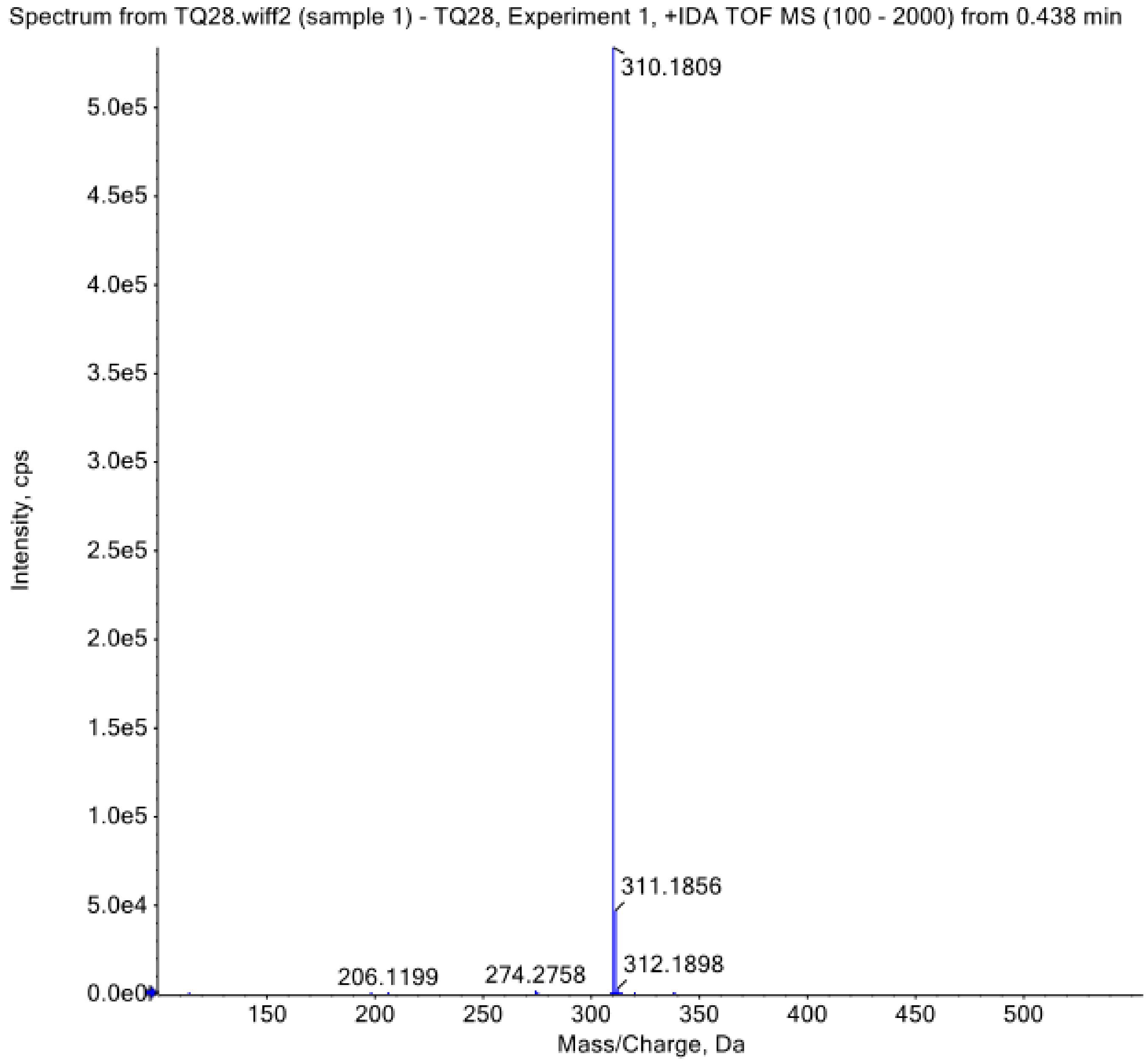

Figure S2. Mass spectra (MS) of TQFL28. High-resolution mass spectra (HR-MS) are applied for monitoring TQFL28. Note: TQ28, TQFL28.
